# 3-Nitrotyrosine Modified Proteins in Atherosclerosis

**DOI:** 10.1155/2015/708282

**Published:** 2015-03-01

**Authors:** Leonor Thomson

**Affiliations:** Instituto de Química Biológica, Facultad de Ciencias, Universidad de la República, Iguá 4225, 11400 Montevideo, Uruguay

## Abstract

Cardiovascular disease is the leading cause of premature death worldwide, and atherosclerosis is the main contributor. Lipid-laden macrophages, known as foam cells, accumulate in the subendothelial space of the lesion area and contribute to consolidate a chronic inflammatory environment where oxygen and nitrogen derived oxidants are released. Oxidatively modified lipids and proteins are present both in plasma as well as atherosclerotic lesions. A relevant oxidative posttranslational protein modification is the addition of a nitro group to the hydroxyphenyl ring of tyrosine residues, mediated by nitric oxide derived oxidants. Nitrotyrosine modified proteins were found in the lesion and also in plasma from atherosclerotic patients. Despite the fact of the low yield of nitration, immunogenic, proatherogenic, and prothrombotic properties acquired by 3-nitrotyrosine modified proteins are in agreement with epidemiological studies showing a significant correlation between the level of nitration found in plasma proteins and the prevalence of cardiovascular disease, supporting the usefulness of this biomarker to predict the outcome and to take appropriate therapeutic decisions in atherosclerotic disease.

## 1. Introduction

A wide range of studies support the role of oxidative stress in the development of cardiovascular disease [1–6], and the evaluation of oxidant-mediated biomolecule modifications is able to predict clinical outcomes [7–9]. The atheromatous process is related to endothelial dysfunction, and the presence of atherosclerotic risk factors such as hypercholesterolemia and hypertension induces the expression of cell adhesion molecules such as VCAM-1, ICAM-1, E-selectin, and P-selectin [10], which promote the adhesion of monocytes and T cells to the vascular endothelium and its transmigration into the subendothelial space. Leukocytes migrating from the blood stream to the vascular wall play a fundamental role in atherosclerosis, acting as nucleating centers for modified biomolecules and also as the main source of oxidants inside the inflamed blood vessel. Uncontrolled uptake of LDL and altered cholesterol efflux are the main factors that contribute to macrophages lipid overload and foam cell formation [11]. In macrophages, the uptake of oxidized LDL is mediated by a group of receptors, including the scavenger receptors class A (SR-A) and CD36, a class B receptor, and the lectin-type oxidized LDL receptor 1 (LOX-1) [12, 13]. On the contrary, the scavenger receptor B1 (SR-B1) and the ATP-binding cassette transporters A1 (ABCA1) and G1 (ABCG1) are responsible for cholesterol efflux [14].

Activation of inflammatory cells into the subendothelial space is tightly associated with generation of reactive oxygen species (ROS) and nitrogen species (RNS), which can mediate protein and lipid modifications. Protein nitration is a posttranslational modification caused by nitric oxide (^•^NO) derived oxidants that frequently modifies the activity of the target molecule [15, 16]. The presence of proteins bearing the 3-nitrotyrosine modification was described in both plasma and atherosclerotic lesions from coronary artery disease patients and also from atherosclerotic prone mice [17, 18].

## 2. Mechanisms of Protein Nitration

**Figure 1:**
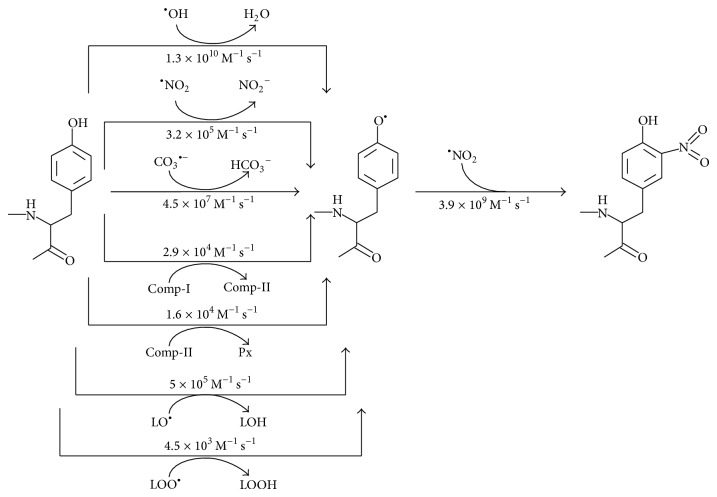
Mechanism of protein 3-nitrotyrosine formation.

Protein nitration involves two steps (Figure 1); in the first one hydrogen atom is lost from the phenolic ring of tyrosine residues with the transient formation of a tyrosyl radical (Tyr^•^). This step is followed by the diffusion controlled reaction of Tyr^•^ with nitrogen dioxide radical (^•^NO_2_) at diffusion controlled rate (*k* = 3.9 × 10^9^ M^−1^ s^−1^) [19] (Figure 1). The initial oxidation of tyrosine can be achieved by a number of oxidants, including hydroxyl radical (^•^OH, *k* = 1.3 × 10^10^ M^−1^ s^−1^) [20] and ^•^NO_2_ (*k* = 3.2 × 10^5^ M^−1^ s^−1^) [21].

Peroxynitrite (ONOO^−^), the diffusion controlled reaction product between ^•^NO and superoxide (O_2_^•−^) (1), generates both radicals [16]:

(1) NO•+O2•−⟶ONOO−

Actually, the homolytic decomposition of the protonated form, peroxynitrous acid (pKa = 6.8), generates ^•^OH and ^•^NO_2_:

(2) ONOO−+H+⟶ONOOH⟶OH•+NO2•

Carbonyl radicals (CO_3_^•−^), produced by decomposition of nitrosoperoxycarboxylate (ONOOCO_2_), the product of the reaction between peroxynitrite and CO_2_(3), also react with tyrosine residues (*k* = 4.5 × 10^7^ M^−1^ s^−1^) [22]:

(3) ONOO−+CO2⟶ONOOCO2⟶NO2•+CO3•−

Lipid-derived alkoxyl (LO^•^) (*k* = 5 × 10^5^ M^−1^ s^−1^) [23] and peroxyl radicals (LOO^•^) (*k* = 4.5 × 10^3^ M^−1^ s^−1^) [24] can also promote one-electron oxidations of tyrosine residues in proteins. Meanwhile, myeloperoxidase (MPO) is able to feed both steps. In the first one, MPO-derived compounds I (*k* = 2.9 × 10^4^ M^−1^ s^−1^) [25] and II (*k* = 1.57 × 10^4^ M^−1^ s^−1^) [26] react with tyrosine to yield Tyr^•^. In addition, both compounds generate ^•^NO_2_ [27] ((4)–(6)), which is able to mediate the modification of tyrosine residues attained in both steps (Figure 1):

(4) $$\begin{array}{c} \text{MPO}+{\text{H}}_{2}{\text{O}}_{2}⟶\text{Comp-I}+{\text{H}}_{2}\text{O} \end{array}$$

(5) Comp-I+NO2−⟶Comp-II+NO2•

(6) Comp-II+2H++NO2−⟶MPO+H2O+NO2•

## 3. Vascular Sources of Reactive Species

Protein tyrosine nitration is localized within specific subcellular compartments in close proximity to the enzymes related to the production of the involved oxidants, as demonstrated by immunoelectron microscopy [28]. The formation of the main precursors of ROS is catalyzed by a group of specially committed enzymes present fundamentally in the plasma membrane and membrane surrounded organelles known as NADPH-oxidase (Nox, EC 1.6.3.1). The Nox family of enzymes is specifically dedicated to generate oxygen derived oxidants, in particular O_2_^•−^ and less frequently hydrogen peroxide (H_2_O_2_). Four Nox isoforms have been found in the vasculature, Nox1, Nox2, Nox4, and Nox5, and at the vascular level Nox enzymes have emerged as the major source of ROS [29]. The different Nox isoforms accomplish several biological functions, which are dependent not exclusively on the enzyme but also on the specific cell type. Nox1 is expressed in endothelium, smooth muscle cells, and adventitial fibroblasts [30, 31], while Nox2, Nox4, and Nox5 are found in all vascular wall cells [31, 32]. Generation of ROS by phagocytic cells, mostly mediated by Nox2, is activated by several stimuli through receptor-mediated protein kinase activation. In fact, cytokines and modified-LDL are able to trigger p47phox phosphorylation and its migration to the plasma membrane where it associates with the electron transferase (gp91phox) and p22phox, activating O_2_^•−^ production. During membrane migration p47phox is escorted by several cytosolic subunits, in particular p67phox and Rac2 [33]. While Nox1 is activated in an analogous way as Nox2, Nox4 activation requires the association of Poldip2 with p22phox [34], and Nox5 is activated by association of Ca^2+^ with its N-terminal calmodulin-like domain, which contains four Ca^2+^-binding EF-hand motifs. As other Nox enzymes, Nox5 is also regulated by protein kinase C (PKC) as well as the tyrosine kinase c-Abl [35].

Other putative sources of ROS at vascular level are xanthine oxidase (XO, EC 1.17.3.2) and the mitochondrial electron transport chain. Unlike its precursor xanthine dehydrogenase (XDH, EC 1.17.1.4), which uses NAD^+^, xanthine oxidase uses oxygen as electron acceptor [36–38]. Superoxide is generated as a mitochondria byproduct, by electron leakages predominantly at complexes I and III [39, 40].

Meanwhile ^•^NO, the main precursor of reactive nitrogen species (RNS) is generated by the family of nitric oxide synthases (NOS, EC 1.14.13.39) from L-arginine. In the vasculature ^•^NO produced by the endothelial isoform or NOS3 is responsible for the endothelial-mediated vascular relaxation. In the vascular wall the inducible form or NOS2 generates ^•^NO after cell stimulation. Macrophage activation may lead to the simultaneous production of O_2_^•−^ and ^•^NO and consequently to ONOO^−^ formation. Actually, the presence of proinflammatory cytokines, as interleukin-1*β*, tumor necrosis factor *α*, and interferon *γ*, generated by inflammatory cells induces simultaneously the assembly of Nox2 and the expression of NOS2 [41–43].

Myeloperoxidase (EC 1.11.2.2) is a member of the mammalian heme peroxidase superfamily of enzymes and uses H_2_O_2_ to form more reactive oxidant species. In the presence of MPO and H_2_O_2_, hypochlorous acid (HOCl) and ^•^NO_2_ are formed from Cl^−^ and NO_2_^−^, respectively [44, 45]. Circulating neutrophils, monocytes, and some tissue macrophages express MPO [46]. While MPO and its products are important defense factors against invading microorganisms, different evidences suggest that excessive activity of MPO can play a role in inflammatory tissue injury. In fact, plasma MPO independently predicted the early risk of myocardial infarction, as well as the risk of other major adverse cardiac events (MACE) in patients with chest pain [47, 48]. Additional evidences of the role of MPO in vascular pathology come from population studies, where elevated circulating levels of this enzyme in an initially healthy population predicted the risk of future coronary heart disease [9, 49, 50]. In patients being treated for coronary artery disease, increased MPO concentrations remained significantly associated with incident MACE over a follow-up of 3 years, even after adjusting for traditional cardiac risk factors, creatinine clearance, B-type natriuretic peptide, and high-sensitivity C-reactive protein [51]. In fact, the accumulation of leukocytes containing MPO in the subendothelial space in sites of erosion and breakdown of coronary plaque has been reported [52, 53], pointing to this enzyme as one of those responsible for the acute coronary syndrome.

## 4. Main Nitration Targets in Atherosclerosis

State-of-the-art technology has allowed precise evaluation of circulating nitrotyrosine modified proteins. Higher levels of 3-nitrotyrosine in plasma proteins have been reported in atherosclerotic patients and accurately measured by stable isotope dilution HPLC with on-line electrospray ionization tandem mass spectrometry (LC/ESI/MS/MS) [54] and specific ELISA techniques developed to measure fibrinogen and 3-nitrotyrosine modified fibrinogen [55, 56]. An important increase of 3-nitrotyrosine modified apolipoprotein A-1 (apoA-1), apolipoprotein B-100 (apoB-100), and fibrinogen has been reported in plasma from individuals diagnosed with coronary artery disease (Table 1). In addition, using specific enrichment and mass spectrometric techniques the site of nitration was identified in several proteins isolated from human plasma (Table 2).

Apolipoprotein A-1, the major protein in high density lipoprotein (HDL), was a preferential target for nitration in subjects with CVD. Experimental evidences support the role of MPO in circulating apoA-1 nitration [57]. In particular, coimmunoprecipitation experiments proved the presence of circulating apoA-1/MPO complexes in HDL isolated from human CVD plasma [58]. Colocalization of 3-nitrotyrosine modified HDL with MPO in human aortic atherosclerotic intima was also reported [59]. Moreover, MPO levels predicted accelerated progression of coronary atherosclerosis in diabetic patients [60]. Consequently, MPO appears to be responsible for the dramatic increase of nitrotyrosine and chlorotyrosine observed within apoA-1 in HDL recovered from serum and atherosclerotic lesions from individuals with CVD. In cholesterol-loaded murine macrophages, nitration and chlorination of apoA-1, both* in vitro* and* in vivo*, resulted in a less effective protein than the unmodified one to stimulate ABCA-1-dependent cholesterol efflux [57, 58, 61]. While in CVD patients, the site of union to lecithin cholesterol acyltransferase (LCAT), involving tyrosine 166, was the primary target for apoA-1 modification (Table 2). Nitration of this tyrosine residue decreased apoA-1-mediated LCAT activation and resulted in a dysfunctional HDL particle [62–64]. MPO-modified apoA-1 showed also a reduced capacity to stimulate endothelial cell proliferation and migration, through decreased Akt and ERK1/2 phosphorylation [65]. The modification of apoA-1* in vitro* by MPO-derived hypochlorous acid, in protein residues different from tyrosine, switched the role of HDL in inflammation from anti- to proinflammatory. In fact, the association of this oxidized lipoprotein form to endothelial cells led to NF-*κ*B activation and the appearance of VCAM on the cell surface. This gain of function was mediated by the saturable and specific binding of oxidized HDL to an unknown endothelial cell receptor, different from the scavenger receptors CD36 and SR-A [66].

Apolipoprotein B-100, the main LDL protein, has also been found nitrated in CVD plasma (Table 1). Several LDL nitration sites were identified by mass spectrometry after authentic peroxynitrite exposure [67, 68] and also by upregulation of Nox expression by bovine aortic endothelial cells exposed to oscillatory and pulsatile shear stress [67]. A similar pattern of tyrosine nitration was observed in circulating LDL isolated from healthy blood donors (Table 2) [69]; no data on CVD patients were reported. Nitrated apoB-100 showed profound conformational changes, which promoted increased LDL binding and uptake by endothelial cells. Internalization of this modified form of LDL was mediated by LOX-1, CD36, and SR-A [69]. Moreover, the* in vitro* exposure to nitrating agents derived from monocytes in the presence of exogenous NO_2_^−^ converted LDL into a form that was taken up and degraded by macrophages, leading to foam cell formation [70].

Fibrinogen is another important target of reactive species in CVD, and increased levels of nitrated fibrinogen were found in patients with coronary artery disease (Table 1) [56]. In otherwise healthy humans, an inflammatory challenge was able to induce fibrinogen nitration [71]. Moreover, in atherosclerosis-prone mice, knockout for the LDL receptor and apolipoprotein B mRNA editing enzyme (apobec), the lack of apoA-1 increased the level of nitrated fibrinogen in plasma, pointing to a subrogate role for the coagulation protein as a nitration target [17]. Besides, cigarette smoking, an important risk factor for both atherosclerosis and thrombosis, also induced an important increase in the level of 3-nitrotyrosine modified fibrinogen. In fact, nitrated fibrinogen was significantly higher in smokers (51.0 ± 5.5 *μ*mol NO_2_-Tyr/mol Tyr) compared with nonsmokers (36.0 ± 3.2 *μ*mol NO_2_-Tyr/mol Tyr) [55]. The presence of 3-nitrotyrosine in fibrinogen *β* chain significantly accelerated fibrin clot formation [17]. The fibrin clot architecture was altered, with increased stiffness, and the rate of clot lysis was reduced by nitration [55, 72]. This modified form of fibrinogen could favor fibrin deposition onto atherosclerotic plaques and explain the increased propensity for thrombotic events found in coronary artery disease subjects and atherosclerosis-prone mice.

To add to the pathogenic effects of protein nitration in atherosclerosis, the presence of increased levels of nitrotyrosine in circulating proteins and proteins isolated from atherosclerotic plaques was associated with the presence of circulating specific immunoglobulins against the nitrated epitope. Anti-3-nitrotyrosine antibodies were strongly associated with angiographic evidence of significant coronary artery disease [18]. The levels of immunoglobulins that recognize 3-nitrotyrosine were significantly high also in plasma of subjects with acute lung injury [73] and in atherosclerosis-prone mice [17]; however the functional repercussion of this immune response still remains unexplored. Passive immunization of experimentation animals would help to understand the role of anti-nitrotyrosine antibodies in atherosclerosis. In addition, the identification of protein(s) responsible for triggering the adaptive immune response against the nitrated epitope would help to bring some light onto the pathways linking inflammation, oxidative posttranslational protein modifications, and atherosclerosis.

## 5. Concluding Remarks

We and others have demonstrated the presence and structural and functional consequences of the modification by 3-nitrotyrosine on plasma as well as tissue proteins [16]. Despite the fact of significant increases observed on protein nitration in CVD, the inhibitory effect of this posttranslational protein modification on the activity of the entire population of protein molecules is minimal, since for each protein molecule modified by nitration there are several hundreds of them still unmodified and active [74]. For instance, apoA-1 nitration increased 47% in CVD patients (Table 1); this supposed a change in the number of tyrosine residues modified by nitration from one residue each 325 protein molecules to one residue each 227 protein molecules. The highly significant number of unmodified protein molecules would be enough to fulfill the protein function without any manifest biochemical effect. However, as already discussed, nitration of apoB-100 and fibrinogen promote new proatherogenic and prothrombotic functions, which together with the onset of an adaptive immune response triggered by the nitrated epitope agree with epidemiological results demonstrating a significant correlation between plasma 3-nitrotyrosine levels and higher cardiovascular risk and support the usefulness of this posttranslational protein modification as a risk marker [75].

## Figures and Tables

**Table 1:** Quantitative estimation of 3-nitrotyrosine in CVD plasma.

	Control		CVD		Change (%)	Reference
	NO_2_-Tyr/Tyr (*μ*mol/mol)^1^	NO_2_-Tyr/protein molecule^2^	NO_2_-Tyr/Tyr (*μ*mol/mol)^1^	NO_2_-Tyr/protein molecule^2^		
Total serum proteins^3^	6.1 (3.9–7.8)	NA	9.0 (5.7–12.9)	NA	47	[57]
ApoA-I^3^	438 (335–598)	1/325	629 (431–876)	1/227	44	[57]
ApoB-100^3^	4.0 (1.3–6.9)	1/1,644	8.7 (5.2–12.1)	1/756	117	[57]
Fibrinogen^4^	24.6 (23.4–25.9)	1/303	31.8 (28.7–34.9)	1/235	29	[56]

^1^Nitrotyrosine levels are reported as median (IQR).

^2^The number of Tyr modified residues per protein molecule (NO_2_-Tyr/protein molecule) was calculated using 153, 7, and 134 Tyr residues for apoB-100, apoA-1, and fibrinogen, respectively, from the PromParam tool (Expasy) [76].

^3^Data in reference [57] were obtained using stable isotope dilution LC/ESI/MS/MS.

^4^Plasma concentrations of total and nitrated fibrinogen in reference [56] were determined by ELISA and reported as mg/mL for fibrinogen and nM for nitrotyrosine; *μ*mol NO_2_-Tyr/mol Tyr were calculated using a molecular weight for fibrinogen of 340 kDa and 134 Tyr residues.

**Table 2:** Nitration sites in plasma proteins identified by mass spectrometry.

Protein	Nitrated Tyr	Effect	References
ApoA-1^1^	Y_166 _and Y_192_	Decreased activation of LCAT and ABCA1	[59, 77–79]
ApoB-100^2^	Y_276_, Y_583_, Y_666_, Y_720_, Y_2524_, Y_3139_, Y_3295_, Y_3489_, Y_4141_	Increased affinity for LOX-1, CD36, SR-A	[69]
Fibrinogen *β* chain^1^	Y_292,_ Y_422_	Accelerated clot formation	[55]
Ig gamma-1 chain C region^1^	Y_161,_ Y_290_	Unknown	[18]
Ig kappa chain C region^1^	Y_32_, Y_84_	Unknown	[18]
Ig lambda chain C region^1^	Y_84_	Unknown	[18]
Ig mu chain C region^1^	Y_276_	Unknown	[18]
Ig heavy chain V-III^1^	Y_33, _Y_80,_ Y_95_	Unknown	[18]
Zinc finger and BTB domain-containing protein 1^1^	Y_83_	Unknown	[18]
Protein EFR3 homolog B^1^	Y_669_	Unknown	[18]

^1^Data from proteins immunocaptured from individuals diagnosed with CVD.

^2^Data from an electronegative LDL fraction isolated from plasma from healthy humans.

## Abbreviations

ROS: Reactive oxygen species
RNS: Reactive nitrogen species
NO_2_-Tyr: 3-Nitrotyrosine
LDL: Low-density lipoprotein
HDL: High density lipoprotein
apoB-100: Apolipoprotein B-100
apoA-1: Apolipoprotein A-1
SR-A: Scavenger receptor class A
CD36: Scavenger receptor CD36
LOX-1: Lectin-type oxidized LDL receptor 1
SR-B1: Scavenger receptor B1
ABCA1: ATP-binding cassette transporter A1
ABCG1: ATP-binding cassette transporters G1
Nox: NADPH-oxidase
MPO: Myeloperoxidase
NOS: Nitric oxide synthase
LC/EIS/MS/MS: Liquid chromatography electron ionization spray mass spectrometry.
